# The relationships between depression and other outcomes of chronic illness caregiving

**DOI:** 10.1186/1472-6955-4-3

**Published:** 2005-02-22

**Authors:** Pao-Feng Tsai, Mary M Jirovec

**Affiliations:** 1 College of Nursing, University of Arkansas for Medical Sciences, Little Rock, Arkansas, USA; 2 College of Nursing, Wayne State University, Detroit, Michigan, USA

## Abstract

**Background:**

Many caregivers with chronically ill relatives suffer from depression. However, the relationship of depression to other outcomes of chronic caregiving remains unclear. This study tested a hypothesized model which proposed that hours of care, stressful life events, social support, age and gender would predict caregivers' outcomes through perceived caregiver stress. Depression was expected to mediate the relationship between perceived stress and outcomes of chronic caregiving (physical function, self-esteem, and marital satisfaction).

**Methods:**

The sample for this secondary data analysis consisted of 236 and 271 subjects from the Americans' Changing Lives, Wave 1, 1986, and Wave 2, 1989, data sets. Measures were constructed from the original study. Structural equation modeling was used to test the hypothesized model, and an exploratory structural modeling method, specification search, was used to develop a data-derived model. Cross-validation was used to verify the paths among variables.

**Results:**

Hours of care, age, and gender predicted caregivers' outcomes directly or through perceived caregiver stress (p < .01). Depression mediated the relationship between perceived stress and psychological outcomes and explained 40% and 11% of the variance in self-esteem and marital satisfaction, respectively.

**Conclusion:**

Depression predicted psychological outcomes. Whether depression predicts physical health outcomes needs to be further explored.

## Background

It is estimated that 31–55% of caregivers of chronically ill elderly relatives experience depression [1], and depression is likely to be one of the first [2] and most enduring psychological outcomes for caregivers [3,4]. Caregivers' depression scores have been found to be substantially higher than those of the general population [1,5,6], and higher levels of caregiving stress have been related to greater depression [7,8] and to more depressive symptoms in caregivers [9].

Factors that may be related to stress and depression in caregivers include hours of care, stressful life events, social support, age, and gender. Studies have found that hours of care were significantly related to caregivers' anxiety/depression and somatic symptoms [10,11], and to their emotional and physical strain [12]. Though stressful life events have been associated with both psychological wellbeing [13,14] and physical symptoms among the general population [14], no studies have examined stressful life events in combination with caregiving stress. Stressful life events might have additional impact on caregivers' health other than chronic caregiving.

Social support may enhance the ability of the individual to cope with events or change the individual's cognitive appraisal of events [15]. Quayhagen and Quayhagen found that caregivers who reported needing more social support had lower well-being scores than other caregivers [16]. In other studies, low social support predicted higher perceived burden [7], and adverse social contacts were associated with increased stress [17].

Age has been shown to have indirect effects on depression through its influence on perceived stress, the coping process, and perceived efficacy [18]. Younger caregivers experience more distress than older caregivers [19,20], and they express more subjective burden than older caregivers [21]. Gender has also been shown to have effects on depression. Female caregivers report more distress [1,22] and higher psychiatric morbidity [5] than male caregivers.

Tsai et al. have suggested that stress and depression are emotional aspects of coping mechanisms and depression is the outcome of perceived caregiver stress [23]. Stress has in turn been shown to be the strongest predictor of depression in caregivers [24]. Though depression has been associated with caregivers' physical health [25,26], the data on physical health are less consistent than on psychological health. Some studies have found that caregivers had poorer self-reported health than non-caregivers [27-29], more chronic illnesses [30], and lower immune function [31]; and they used more health care services and took more prescriptions [27]. Convinsky et al. reported that depression was associated with physical function dependence. Caregivers with functional dependence has 2.53-fold chance to be depressed as compared to those who with functional independence [32]. Other studies, however, have found that caregivers did not use more medical services [33] or rate their physical health as less satisfactory than the general population [6]. Further, as Schulz, Visintainer and Willamson point out, even though some studies have suggested possible effects of caregiving on physical health, the evidence is confounded by sampling bias, inadequacy of measurements, and subjective appraisals [34].

Although the associations between depression and self-esteem and marital satisfaction have been examined extensively, only a few studies have been conducted in the context of caregiving. Caregivers have been shown to have lower self-esteem [35], and this has been associated with depressive symptoms [10]. Caregivers who had higher self-esteem experienced less depression [36]. In one study, depressed caregivers were more likely to experience less marital satisfaction [37]. Also, high levels of marital conflict were associated with high levels of depression in adult daughter caregivers [38]. Finally, spousal caregivers reporting low marital cohesion and satisfaction had more depressive symptoms [39].

A recently developed Theory of Caregiver Stress [23] based on theoretical propositions from the Roy adaptation model [40] suggests that depression is the mediator between perceived stress and self-esteem and marital satisfaction. However, the relationships of depression to other outcomes of caregiving, such as physical function, self-esteem and marital satisfaction, remain unclear. The research reported here therefore explored these relationships. We proposed that hours of care would be the primary source of caregiver stress. Stressful life events, social support, age, and gender were antecedent variables and expected to influence caregivers' outcomes through caregiver stress. Depression was conceptualized as a mediator between caregiver stress and other outcomes of chronic caregiving (physical function, self-esteem, and marital satisfaction). Thus, a high level of stress was expected to lead to a high level of depression, which in turn would result in lower levels of physical function, self-esteem, and marital satisfaction.

## Methods

### Sample

Data for the study were obtained from the Americans' Changing Lives (ACL) Survey: Wave 1, 1986 (N = 3,617), and Wave 2, 1989 (N = 2,867) [41]. The ACL collected longitudinal data on subjects aged 25 years and over in the continental United States. Individuals residing in group homes or institutions were not included. The survey used multistage-stratified probability sampling, with Blacks and elderly (60 years and older) oversampled. We used Wave 2 data to test hypotheses and build a data-derived model. Wave 1 data were then used for model validation.

Only individuals with experience in caregiving to a chronically ill aged relative were included in the analyses reported here. This reduced the number of cases available for study to 335 from Wave 1 and 271 from Wave 2. The two samples were not completely independent because 99 cases were included in both waves; therefore, to ensure the independence of samples, these 99 cases were dropped from Wave 1. The final samples from Wave 1 and Wave 2 were thus 236 and 271, respectively.

### Measures

Since the study was a secondary data analysis, indicators of the study variables (hours of care, age, gender, social support, stressful life events, perceived stress, depression, physical function, self-esteem and marital satisfaction) were selected from the Americans' Changing Lives Survey questionnaires, based on face validity. That is, the questions selected gave the appearance of measuring the content of interest. Exploratory factor analysis, confirmatory factor analysis, and internal consistency tests were then conducted to confirm the underlying structures of established scales and develop outcome measures for the current study.

Hours of care were the total hours estimated by the caregiver in the past year, categorized as less than 20 hours, 20 to 39 hours, 40–79 hours, 80–159 hours, and 160 hours or more. Providing more hours of care was expected to indicate more burden of caregiving.

Stressful life events were measured by a 12-item checklist of negative or undesirable events, such as being robbed or burglarized, losing a job, being physically attacked, or experiencing the death of spouse, death of a parent, death of a close friend/relative, serious illness, life-threatening illness/accident, divorce/separation, serious financial problem, death of children, and other such events. Respondents were asked to report whether they had experienced any of these events within the past 2 years. A simple score, the stressful life events index, was created by summing the number of events reported by each respondent. A high score reflected more stressful life events.

Social support was measured by two items: friends/relatives' love and care, and their willingness to listen. Alphas reliabilities were .73 and .74 for Wave 1 and Wave 2, respectively. Higher scores indicated greater support from friends/relatives. Demographic data included age, defined as the chronological age of the caregiver, and gender, coded as biological sex identity.

Perceived caregiver stress was measured by one item asking how much stress the caregiver felt about caring for or arranging care for the elderly relative. Responses were on a 5-point scale ranging from not stressful to very stressful; a higher score reflected more perceived stress. Other studies have shown that stress was associated psychosocial well-being, such as depression [42,43]; in this study the correlation between perceived caregiver stress and depression was .25 (p < .001).

Depression was measured by the 11-item Center for Epidemiological Studies Depression (CES-D) scale [44], which assesses mood and level of overall functioning in the last 7 days. The CES-D was originally developed as a 20-item unidimensional scale. The shorter 11-item CES-D version contains items on feeling depressed, restless, happy, lonely and sad; feeling that people dislike me; people are unfriendly; I enjoy life (reverse scored); I have a poor appetite; cannot keep going; and everything is an effort. The items are rated on a 3-point scale from "hardly ever" to "most of the time." Higher scores indicate higher levels of depression. Based on exploratory factor analysis, three factors of the CES-D scale – depressed and positive mood, somatic symptoms and interpersonal relations – were identified as indicators of the latent variable, depression

Physical function was defined as consisting of functional health, number of chronic illnesses, and self-rated health. Functional health was measured by asking the caregiver whether the caregiver was bedbound, and whether the caregiver had difficulty bathing, climbing stairs, walking, or doing heavy housework, and the degree of difficulty of these tasks. Higher scores reflected a higher level of physical function. The number of chronic illnesses was the sum of the following: arthritis or rheumatism, lung disease, hypertension, heart disease, diabetes, cancer, circulation problems, stroke, fracture, and urinary incontinence. A low score on this measure indicated high physical function. Self-rated health was measured by a single item that asked caregivers to rate their own health on a 4-point scale ranging from poor to excellent. A high score reflected high physical function.

The caregiver's self-esteem was measured by five items: "I take a positive attitude toward self," "I am no good at all," "I see myself as a failure," "I have the feeling of being pushed around in life," and "I perceive myself able to solve problems." These items were measured on a 4-point scale ranging from strongly agree to strongly disagree. A higher score indicated higher self-esteem.

Marital satisfaction was also measured by five items: "Overall satisfaction with relationship," "love and affection expressed from spouse or significant other," "spouse treats me well," "thinking about divorce or separation," and "things happened that I can never forget." Higher scores indicated more marital satisfaction.

Cronbach's alphas for all measures were above the acceptable criterion of .70 in both waves except for self-esteem in Wave 1. However, that measure was on the margin of acceptance, at .68. Since Cronbach's alpha is a conservative estimate of internal consistency [45], the self-esteem index was retained.

### Analytic procedure

Univariate and bivariate analyses were used to examine the descriptive findings. To test the appropriateness of the indicators for each latent variable in both waves, the following procedures were used. First, a single indicator was extracted when applicable (e.g., for social support, self-esteem, and marital satisfaction), and summary scale scores were used as single indicators. This strategy was used to reduce the number of parameter estimations in a complex model; it is considered appropriate when individual factor item loadings in a specific scale are high [46]. Second, for all latent variables with single indicators (i.e., hours of care, stressful life events, social support, age, gender, perceived caregiver stress, self-esteem, and marital satisfaction), the measurements were assumed to be perfect (with 0% error). This conservative estimation was made since increasing measurement errors would induce artificial correlations among the latent variables in the measurement model. Thus, a full factorial loading of 1.0 was assumed for all single indicators in the subsequent latent variables. For latent variables with multiple indicators (i.e., depression and physical health), one factor loading was arbitrarily set to 1.0 to test the relative contribution of the factors. Error variances were not allowed to correlate, but all the latent variables were allowed to correlate with each other. The confirmatory factors analysis indicated that all factor loadings were above 0.4 and significant (p < .01), and they accounted for at least 16% of the true score variance [47]. The only exception was the "interpersonal" factor in depression, with a factor loading of 0.39. Although it was slightly below the required value of 0.4, it was included because it is a well established measure of depression. The factor loading and measurement error for each indicator are shown in Table 1.

**Table 1:** Standardized factor loadings and measurement error variances for the measurement model predicting caregiver stress

Latent variable	Indicators	Factor loading (Measurement error)
Hours of care	Hours of care	1.00^a ^(.00)^b^
Stressful life events	Number of stressful life events	1.00^a ^(.00)^b^
Social support	Friend/relatives positive support	1.00^a ^(.00)^b^
Age	Age	1.00^a ^(.00)^b^
Gender	Gender	1.00^a ^(.00)^b^
Perceived stress	Perceived caregiver stress	1.00^a ^(.00)^b^
Depression	CES-D Depressed & positive mood	.75^a ^(.44)
	CES-D Somatic symptoms	.74 (.45)
	CES-D Interpersonal	.39 (.84)
Physical function	Functional health	.55^a ^(.70)
	Numbers of chronic illness	.65 (.57)
	Self-rated health	.77 (.41)
Self-esteem	Self esteem/mastery index	1.00^a ^(.00)^b^
Marital satisfaction	Marital satisfaction index	1.00^a ^(.00)^b^

Factors and measurement errors were from the completely standardized solution. All factor loadings and measurement errors were significant at .01 level in the preliminary measurement model.

^a ^Parameter was fixed to 1.0 in the unstandardized solution.

^b ^Parameter was fixed to 0 in the unstandardized solution.

**Table 2:** Sample characteristics and comparisons by waves

	Wave 1			Wave 2			
					
Measure	n (236)	%	M(SD)	n (271)	%	M(SD)	p^a^
Hours of care							
<20 hours	38	16.1		30	11.1		.45
20–39 hours	28	11.9		39	14.4		
40–79 hours	35	14.8		48	17.7		
80–159 hours	35	14.8		42	15.5		
≥160 hours	100	42.4		112	41.3		
Stressful life events			.57 (.69)			.50 (.64)	.13
Social support			7.65 (1.95)			7.91 (1.77)	.12
Age, in years			53.56 (16.36)			53.41 (14.43)	.91
Gender							
Male	85	36.0		86	31.7		.31
Female	151	64.0		185	68.3		
Perceived caregiver stress							
Not at all stressful	46	19.5		57	21.0		.75
Not too stressful	62	26.3		78	28.8		
Somewhat stressful	77	32.6		76	28.0		
Quite stressful	26	11.0		35	12.9		
Very stressful	25	10.6		25	9.2		
Physical function							
1) Functional health							
Most severe impairment	7	3.0		9	3.3		.87
Moderately severe impairment	19	8.1		17	6.3		
Least severe impairment	18	7.6		23	8.5		
No impairment	192	81.4		222	81.9		
2) Number of chronic illnesses			1.26 (1.28)			1.37 (1.34)	.36
3) Self-rated health							
Excellent	34	14.4		42	15.5		.95
Very good	88	37.3		95	35.1		
Good	65	27.5		79	29.2		
Fair	38	16.1		45	16.6		
Poor	11	4.7		10	3.7		
Self-esteem			16.02 (3.11)			16.84 (2.86)	.00
Marital satisfaction^b^			-0.12 (3.66)			0.05 (3.61)	.67
Depression			16.00 (4.14)			15.15 (3.87)	.02

^a ^Statistical significance is determined either by t-test or chi-square. ^b ^Score of marital satisfaction is standardized.

A covariance matrix derived from data in the Wave 2 sample was analyzed as input data in the process of model testing. Hypothesized models were tested using the maximal likelihood procedure in the LISREL statistics program. The model tests used absolute goodness-of-fit indices (Chi-square [χ^2^], the goodness-of-fit index [GFI], and the adjusted goodness-of-fit [AGFI]) and comparative fit indices (change in Chi-square [Δ χ^2^], the relative noncentral index [RNI] and the relative normed fit index [RNFI]). Values of GFI, AGFI, RNI, and RNFI between 0.90 and 1.00 were considered to indicate a good fit between the model and the data [48].

An exploratory structural modeling method, specification search [49-51], was then used to develop the data-derived model for the Wave 2 sample. The specification search procedure removed all invalid paths in the hypothesized model and added plausible paths suggested by the modification index. Cross-validation was performed to verify that this data-derived model was valid and stable across samples. In this procedure, the data-derived model was cross-validated by the Wave 1 sample, with both Wave 1 and Wave 2 data sets as input files at the same time.

## Results

### Descriptive findings

The characteristics of the two wave samples are summarized in Table 2. Less than half the caregivers spent more than 160 hours per year taking care of their chronically ill relative (42.4%, Wave 1 and 41.3%, Wave 2). About half reported no stressful life events in the past 2 years (51.7%, Wave 1 and 57.6%, Wave 2); 38% in Wave 1 and 35% in Wave 2 reported one stressful life event in the past 2 years, and 10.1% and 7.4% in Waves 1 and 2, respectively, reported two or more such events. There was no significant difference in social support between the two samples, although mean scores on social support were slightly higher in Wave 2 (M [SD] = 7.91 [1.77]) than in Wave 1(M [SD] = 7.65 [1.95). The average age was 53 in both waves. Sixty-four percent of the caregivers in Wave 1 and 68% of those in Wave 2 were female.

Respondents in both waves rated perceived caregiver stress similarly: 21.6% and 22.1% in Waves 1 and 2, respectively, reported "quite and very" stressful experiences, while over 45% in both waves reported that their experiences were not at all stressful or not too stressful.

Caregivers' physical function and marital satisfaction were also similar in both waves. Approximately 81% of the caregivers in each wave reported no functional impairment. Only approximately 20% of caregivers rated their health fair or poor, and the averages numbers of chronic illnesses were 1.26 and 1.37 for Waves 1 and 2, respectively. Scores on caregivers' marital satisfaction were -. 12 and .05 for Waves 1 and 2, respectively; the difference was not significant. However, caregivers in Wave 1 had significantly lower self-esteem scores than those in Wave 2 (16.02 vs. 16.84, p < 0.01). Depression also differed significantly (p < 0.05). Respondents in Wave 1 reported more depression than those in Wave 2, with mean scores on the 11-item CES-D of 16.00 (SD = 4.14) and 15.15 (SD = 3.87) for Waves 1 and 2, respectively.

### The hypothesized model

When the hypothesized model was tested to determine whether depression mediated the relationship between perceived caregiver stress and caregiving outcomes (physical function, self-esteem, and marital satisfaction), the statistics showed a moderate fit between the model and the data (χ^2 ^= 237.22; d.f. = 73; GFI = .89; AGFI = .84; RNFI = .85).

**Figure 1:**
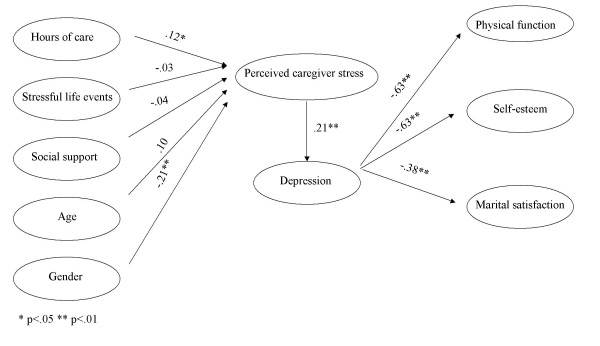
The hypothesized model

More hours of care and female gender predicted greater caregiver stress, accounting for 7% of the variance in stress, as shown in Figure 1. Greater perceived caregiver stress was associated with higher depression. Higher levels of depression in turn predicted poorer physical function, lower self-esteem, and lower marital satisfaction, accounting for 40%, 40%, and 15% of the variance in physical health, self-esteem, and marital satisfaction, respectively. Depression served as a mediator between perceived caregiver stress and caregivers' physical function, self-esteem, and marital satisfaction.

### The data-derived model

Since the hypothesized model fit the data only moderately well, a data-derived model was built to compare with the hypothesized model. After all the insignificant paths in the hypothesized model had been dropped, paths were added at each step to improve the goodness-of-fit statistics based on the modification index and pre-set assumptions. The resulting data-derived model had a good fit with the data (χ^2 ^= 147.73; d.f. = 74; p = .00; GFI = .93; AGFI = .90; RNFI = 1.00), close to that of the measurement model, with an insignificant difference (Δ χ^2 ^= 33.58; Δ d.f. = 34; p = ns).

**Figure 2:**
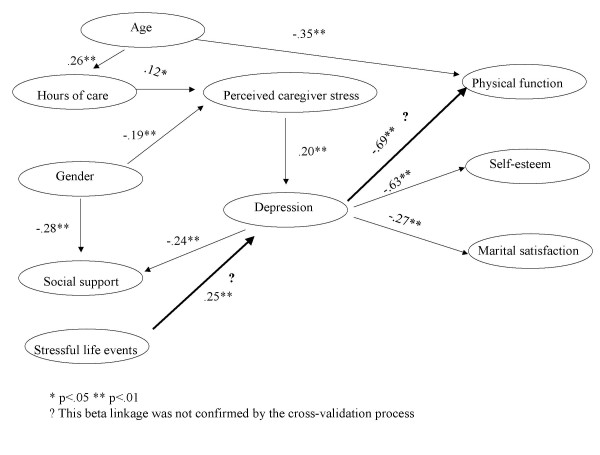
The data-derived model

The strength and direction of the relationships among the latent variables are shown by the standardized coefficients in Figure 2. The data-derived model accounted for 6%, 10%, 57%, 40%, and 11% of the variance in perceived caregiver stress, depression, physical function, self-esteem, and marital satisfaction, respectively. Hours of care were predicted by age: older caregivers provided more hours of care than younger caregivers. Social support was predicted by gender and depression: males and depressed caregivers tended to have less social support. Being a female caregiver and giving more hours of care made the caregiver more susceptible to perceived stress. Having less caregiver stress and fewer stressful life events reduced the chances of depression. Older age and higher levels of depression tended to result in poorer physical function. Greater depression was also associated with less self-esteem and less marital satisfaction. The data-derived model confirmed that perceived stress mediated the relationships between hours of care, gender, and depression, while depression was the mediator between perceived stress and other outcome variables (physical function, self-esteem, and marital satisfaction).

### Cross-validation

In order to test the robustness of the paths across the samples, the data-derived model was cross-validated by Wave 1 sample. The results are summarized in Table 3. The validating process showed the data-derived model was not confirmed only in Step 2 when the paths between endogenous variables were constrained (p < .01). This step showed that some paths between endogenous variables were not confirmed by Wave 1. To investigate the differences between Wave 1 and Wave 2, especially in the Beta linkage, each path was examined individually. The results showed that the differences came from the links between stressful life events and depression, and depression and physical function. That is, the cross-validation procedure confirmed that the data-derived model was stable across two waves of data, except in two paths (stressful life events to depression, and depression to physical function). These two paths need to be further examined.

**Table 3:** Summary of cross-validation for the data-derived model

	Comparison to previous model					
	
Steps and Purpose	χ^2^	df	p	χ^2^_diff_	df_diff_	p
Step 0 Factor loadings, path coefficients, factor variance, and covariance were all set to be inequality across group	369.14	148	.00			
Step 1 Constrain factor loadings	376.21	152	.00	7.07	4	n.s.
Step 2 Constrain factor loadings, and the paths between endogenous variables (beta linkages)	390.92	159	.00	14.71	7	<.01
Step 3 Constrain factor loadings, the paths between endogenous variables (beta linkages), and the paths between exogenous variables and endogenous variables (gamma linkages)	396.04	163	.00	5.12	4	n.s.
Step 4 Constrain factor loadings, path coefficients, and error variance	401.39	169	.00	5.35	6	n.s.
Step 5 Constrain factor loadings, path coefficients, error variance, and factor variance	413.63	179	.00	12.24	10	n.s.

## Discussion

The hypothesized model postulated that hours of care would be the most important factor in perceived caregiver stress, and other factors would include stressful life events, social support, age, and gender. Higher perceived caregiver stress was expected to result in more depression, which in turn would lead to poorer health function, lower self-esteem, and lower marital satisfaction. These expectations were only partially supported by the data.

The data-derived model suggested that age had an indirect effect on perceived caregiver stress, through hours of care. Stone et al. found that older caregivers tended to assume the role of primary caregiver in attending to their chronically ill relatives [28]. Thus, it is likely that the older caregivers in this sample assumed more hours of care than young caregivers, resulting in more caregiver stress.

Age was also found to predict physical function. George and Gwyther noted that spouse caregivers were more susceptible to diminished physical function than other caregivers and this was probably caused by their older age [33].

Stressful life events failed to predict perceived caregiver stress; instead, stressful life events predicted caregivers' outcomes through depression. One explanation for this might be that stressful life events and caregiver stress work independently in predicting caregivers' outcomes. One other study found that stressful life events did not influence health outcomes through perceived stress; rather, they were a confounding factor in predicting health [52]. And Stone et al. reported that stressful life events led directly to adverse health outcomes instead of being mediated by perceived stress [53].

Hours of care and perceived caregiver stress were expected to play important roles in caregiver outcomes. However, while hours of care predicted caregiver stress, hours of care was not the only nor the most important determining factor. Further, perceived caregiver stress explained only a small amount of the variance in depression. This is consistent with Pruchno et al's finding that caregiving had little impact on depression or the physical health of the caregiver [25]. The present findings support the view that caregivers of chronically ill relatives adapt to the demands of the situation and stabilize or even improve over time [54]. It is possible that the chronic nature of the recipient's illness enables the caregiver to adjust to persistent needs and reestablish a balanced life over a period of providing care. Viewed from this perspective, hours of care should not be expected to have a major effect on perceived caregiver stress. It can also be argued that caregivers confront many problems other than caregiving burden, and the impact of chronic caregiving may be diluted by competing daily stressors or stressful life events. Thus, the relationship between hours of care and perceived caregiver stress may not be as clear in long-term caregiving as in short-term caregiving to an acutely ill relative.

Unexpectedly, in this study social support had no impact on perceived caregiver stress; but this is not unprecedented: similar results were reported by Lawton et al. [8]. We used friend/relative positive support as the indicator of social support; however, some aspects of social support may be more important than others in reducing caregiver stress. It is also possible that the measure used here was not sensitive enough to detect actual social support.

Research has shown that low social support makes people more vulnerable to depression, and that has been clearly demonstrated for the elderly [55-58]. However, in our study, depression predicted social support rather than the other way around. Depressed persons may withdraw from some aspects of life, including their social network, especially friends and non-nuclear family relatives.

The data-derived model showed that, as expected, depression mediated the relationship between perceived caregiver stress and self-esteem and marital satisfaction. However, the relationship between depression and physical function was not confirmed by the cross-validation. This is inconsistent with the findings of Pruchno et al. [25] and Zanetti et al. [26]. One possible explanation is that in this sample, depression and physical function may both have been outcomes of important factors that were not included in the study. Clearly, the relationship between depression and physical function needs to be further examined.

The study was limited to the variables in the original Americans' Changing Lives survey, constricting our choices in operationalizing constructs. Further, the study was cross-sectional and consequently was limited in testing the causal relationships depicted in the model. Although the findings provide preliminary evidence of causal relations among the variables, better examination of causality will require longitudinal data.

## Conclusion

In spite of its limitations, the study shows the importance of psychological mediators in the care of a chronically ill relative. The question of how caregivers manage to avoid adverse outcomes or why some caregivers are at risk for adverse outcomes can be answered in part by understanding the role of depression. Clearly, to avoid adverse outcomes, clinical interventions should target caregivers who are experiencing depression.

## List of abbreviations used

Americans' Changing Lives: ACL

Center for Epidemiological Studies Depression: CES-D

The goodness-of-fit index: GFI

The adjusted goodness-of-fit: AGF

The relative noncentral index: RNI

The relative normed fit index: RNFI

## Declaration of competing interests

The author(s) declare that they have no competing interests.

## Authors' contributions

Author 1, PT, developed the research proposal, carried out the data analysis, interpreted the data and participated in the sequence alignment of the manuscript. Author 2, MMJ, participated in developing the research proposal and interpreting the data.

## Pre-publication history

The pre-publication history for this paper can be accessed here:


